# Moral courage and its influencing factor among oncology specialist nurses: A multi‐centre cross‐sectional study

**DOI:** 10.1002/nop2.2096

**Published:** 2024-01-17

**Authors:** Hongling Zheng, Lei Luo, Xiaohui Tan, Yao Cen, Naifang Xing, Hui Huang, Yanling Cai

**Affiliations:** ^1^ Nursing Department Sichuan Clinical Research Center for Cancer Sichuan Cancer Hospital & Institute, Sichuan Cancer Center, Affiliated Cancer Hospital of University of Electronic Science and Technology of China Chengdu China; ^2^ School of Nursing Chengdu Medical College Chengdu China

**Keywords:** cross‐sectional study, influencing factors, moral courage, moral distress, moral sensitivity, oncology specialist nurses

## Abstract

**Aim:**

Moral courage among healthcare workers has been extensively studied. However, few studies have been conducted on oncology specialist nurses, who frequently encounter complex moral situations. This study aimed to describe the current situation regarding moral courage and explored its influence on oncology specialist nurses in China.

**Design:**

This was an exploratory, descriptive study.

**Methods:**

A convenience sample of 390 nurses was conducted from 15 hospitals in Sichuan Province, China, between March and May 2023. Participants were assessed using the Moral Distress Scale‐Revised, Nurses' Moral Courage Scale and the Moral Sensitivity Questionnaire.

**Results:**

The results demonstrated that moral courage was negatively associated with moral distress, and positively associated with moral sensitivity. Having a master's degree or above, an intermediate title or senior title, medical ethics training, moral distress or moral sensitivity contributed to explaining 54.1% of the variance in moral courage.

**Conclusions:**

Moral courage was associated with several factors. Developing clinical intervention strategies and effective teaching methods will be critical for improving moral courage.

No Patient or Public Contribution.

## 1 INTRODUCTION

Cancer is a major public health problem that seriously endangers both public health and social development. According to GLOBOCAN 2020 data, China was estimated to have had more than 4.5 million new cases of confirmed malignant tumours and approximately three million deaths from malignant tumours in 2020 alone, accounting for 23.7% and 30.2% of new malignant tumour diagnoses and deaths worldwide in 2020 respectively (Qiu et al., 2021). Oncology specialist nurses play an important role in cancer prevention, nursing, rehabilitation and health education (Li et al., 2020). Nursing is widely considered an ethical profession that calls on nurses to have the courage to stand up for what they think to be right, to be forceful about it, and to live according to their moral standards (Numminen et al., 2019). However, acting honourably, that is, following one's ideals and convictions, is not always easy (Hauhio et al., 2021). Recent changes in attitudes and underlying philosophies have influenced nursing (Peng et al., 2023). Oncology nursing involves numerous situations related to ethical issues, since a nurse is a person with whom the patient has the most contact during hospitalization. This can lead to ethical conflicts between nurses and healthcare organizations, colleagues and patients (Jun & Wang, 2021).

A moral perspective is implicit in all nursing activities since ethical problems are a component of daily nursing practice (Thompson et al., 2006). There has been a growing focus on nurses' moral courage as a component of their moral competence (Hu et al., 2022). In the field of nursing, moral courage is defined as a nurse's adherence to their professional code of ethics and strict adherence to these guidelines, even if such adherence has a foreseeable or real negative impact on them (Numminen et al., 2017). Moral courage is a fundamental attribute for nurses and is extremely effective in dealing with ethically demanding situations. Moral courage and moral sensitivity have been suggested as significant means to relieve moral distress (Kleemola et al., 2020). Moral sensitivity is a requirement for nurses' professional competency and an appropriate humanistic value in relation to patient care (Grady et al., 2008) as it involves the capacity to identify moral distress in practical circumstances (Lovett & Jordan, 2010). Moral distress arises in those exposed to situations or events that challenge their ethical belief systems and basic values (Ulrich & Grady, 2023). Moral distress, affecting nurses in all healthcare areas, occurs especially in situations where environmental conditions make morally good decisions challenging or impossible, and is regarded as a major issue confronting the nursing profession (Lazzarin et al., 2012). A high level of moral distress has been linked to an elevated risk of post‐traumatic stress disorder, depression, anxiety, compassion fatigue and occasionally, burnout (Bradshaw et al., 2022; Eche et al., 2023; Smallwood et al., 2021).

### 1.1 Background

Moral courage plays a critical role in holistic nursing and varies across regions and populations. A variety of studies have explored the relationship between moral courage, moral sensitivity and moral distress among nurses who care for patients with various kinds of diagnoses (Konings et al., 2022; Pakizekho & Barkhordari‐Sharifabad, 2022; Peng et al., 2023), nursing students (Escolar‐Chua, 2018; Gibson et al., 2020; Huang et al., 2023; Koskinen et al., 2021; Mohammadi et al., 2022), psychiatric nurses (Jiao et al., 2022) and intensive care unit (ICU) nurses (Giannetta et al., 2022; Goktas et al., 2023; Zhang et al., 2023). These studies showed a positive correlation between moral courage and moral sensitivity, and a negative correlation between moral courage and moral distress.

Unlike patients with diagnoses other than cancer, patients with cancer mostly have clear consciousness and good verbal communication throughout the disease course, while suffering from various types of burdens. Oncology health workers must help patients and their families make a large number of decisions, especially in resource‐limited areas (DeBoer et al., 2021). China is a country with 1.4 billion people and medical resources are tight. Oncology specialist nurses are more exposed to situations that cause moral distress than those who provide care to patients with other illnesses (Cohen & Erickson, 2006; Eche et al., 2023). Nevertheless, areas such as oncology, have not been thoroughly explored in studies on ethical issues in nursing, even though ethical dilemmas concerning cancer nursing continue to expand (Lazzarin et al., 2012).

To date, no studies have explored moral courage or its related factors among oncology specialist nurses. A deeper understanding of moral courage and more effective methods for dealing with ethically challenging situations are needed (Lazzarin et al., 2012). To fill this gap in the current research, various multi‐centre studies have been conducted but not in this specialist area of nursing. The purposes of this exploratory, descriptive study were to (1) describe the current status of moral courage among Chinese oncology specialist nurses; (2) explore the relationship between moral courage, moral sensitivity and moral distress and (3) investigate the factors that influence moral courage in the Chinese medical environment.

## 2 METHODS

### 2.1 Design and setting

This cross‐sectional study collected data using an approximately 20‐minute self‐report questionnaire. Information was gathered from 15 tertiary hospitals in Sichuan Province, China, between March and May 2023. These hospitals are located in five different regions of Sichuan Province and confront varying types of medical situations. Each hospital has more than 1000 beds and plays a critical role in treating local patients.

### 2.2 Sampling and recruitment

G*power software (version 3.1.9.6) was used to estimate the minimal sample size. A regression model based on a linear multiple regression analysis with a medium effect size of 0.15, an alpha equivalent to 0.05 and a power (1‐*β*) of 0.95 showed that, with a 20% dropout rate, 143 cases were needed for the study's sample size. Located in southwest China, Sichuan Province has a population of more than 80 million and is the fifth most populated in the country. Comprising 486,000 square kilometres and 21 cities/counties, we divided the province according to its population distribution and economic development into five areas, including Cheng Du (the provincial capital), Western Sichuan, Eastern Sichuan, North Sichuan and South Sichuan. Based on the relationship network of the corresponding author and the extent of access to data, we selected three hospitals in each area using convenience sampling, with 15 tertiary hospitals finally included. In each hospital, a responsible person was designated to work alongside the oncology specialist nurses throughout the hospital and instruct nurses who met the inclusion criteria on how to complete the questionnaire. The inclusion criteria for the participants were as follows: (1) qualified oncology specialist nurses, (2) working in a tertiary hospital and (3) voluntarily participating in the study with informed consent. The exclusion criteria were as follows: nurses enrolled in study programmes, nurses on rotation or nurses on leave for any reason during the survey period.

### 2.3 Measures

#### 2.3.1 Self‐designed socio‐demographic characteristics questionnaire

A self‐designed socio‐demographic characteristics questionnaire was used, which included 16 variables: sex, age, education level, title, marital status, nature of your hospital, hospital level, category of your hospital, position, department, employment form, working experience in nursing, time of obtaining oncology specialist nursing certification, oncology specialist nursing certification level, religious beliefs and medical ethics training.

#### 2.3.2 Moral courage scale

The moral courage scale (MCS) was developed by Numminen et al. (2019) and introduced in China by Wang (Numminen et al., 2019; Wang et al., 2019). This scale includes 21 items that measure nurses' moral courage in four dimensions: compassion and true presence (five items), commitment to good care (five items), moral integrity (seven items) and moral responsibility (four items). Items were assessed on a 5‐point Likert‐type scale, with scores ranging from 1 (does not describe me at all) to 5 (describes me very well). Higher scores indicate greater moral courage. Cronbach's alpha and split‐half coefficients for the Chinese version of the MCS were 0.905 and 0.884 respectively. The content validity index of this scale was 0.910, and its structural validity was evaluated using factor analysis. Exploratory factor analysis extracted four common factors with a cumulative analysis of variance (ANOVA) of 60.567% (Wang et al., 2019).

#### 2.3.3 Moral distress scale

Moral distress was assessed using the moral distress scale (MDS)‐Revised, which is a translated and validated Chinese version of the MDS (Sun et al., 2012; Wang et al., 2019). It assesses four dimensions: individual accountability (eight items), not in the patients' best interests (five items), value conflict (six items) and damage to patients' interests (three items). Participants grade the frequency and intensity of moral distress on a scale ranging from 0 (never) to 4 (very regularly) and from 0 (none) to 4 (large degree) respectively. The total score, which ranges from 0 to 352, is calculated by multiplying the frequency score by the intensity score for each of the 22 items. Higher scores indicate more serious moral distress. With a Cronbach's alpha of 0.879 and a retest reliability of 0.80, the MDS‐Revised has shown strong dependability. The content validity of the scale was 0.909. Factor analysis extracted four main factors that explained 54.583% of the total variance, indicating that the scale had good structural validity (Wang et al., 2019).

#### 2.3.4 Moral sensitivity questionnaire

Lutzen created and revised the moral sensitivity questionnaire (MSQ), and Huang translated it into Chinese and performed reliability testing (Huang et al., 2016; Lützén et al., 2000, 2006). It assesses two dimensions: moral responsibility and strength (five items), and a sense of moral burden (four items). It is graded on a 6‐point Likert scale, with scores ranging from 1 (‘I totally disagree’) to 6 (‘I totally agree’). The total score on this scale ranges from 6 to 54, with a higher score indicating greater moral sensitivity. The Cronbach's alpha coefficient was 0.820, and the split‐half reliability was 0.746, indicating good reliability and validity (Huang et al., 2016). The Kaiser–Myer–Olkin value was 0.825, and the approximate chi‐square for the Bartlett's test was 845.829 (*p* < 0.001). Factor analysis extracted two factors that explained 56.4% of the total variance, indicating good structural validity (Huang et al., 2016).

### 2.4 Data collection

The survey was conducted using the internet data collection tool Questionnaire Star (www.wjx.cn). The corresponding author distributed the link to the survey to those in charge of the selected hospitals between March 2023 and May 2023, after which relevant hospital officials sent the link to their oncology specialist nurses. Participants with identical IP addresses or WeChat IDs could only submit an online survey once to prevent spam. All data, including WeChat IDs and IP addresses, were utilized only for research and were accessible to the researchers. The expected time to complete all survey items was 20 min, based on the total number of items. To guarantee data accuracy, we removed any surveys that were completed in less than 10 min or more than 20 min, leaving 349 valid surveys.

### 2.5 Data analysis

Data were imputed using SPSS18.0 for analysis. Means and standard deviations were used to express constant variables while numbers and percentages were used to express the categorical variables. Participants' demographics were used in univariate analyses to test for relationships with moral courage. Pearson's correlation coefficients were calculated to identify the relationships between moral courage, moral distress and moral sensitivity. The *t*‐test, one‐way ANOVA and LSD tests were used to compare the scores of various groups. In the stepwise multivariate multiple regression analysis, variables with a significance of *p* < 0.05 in the univariate analysis were chosen as potentially independent variables. Dummy codes were set as non‐continuous predictor variables before entering them in the regression.

### 2.6 Ethics statement

Before answering the questionnaires, participants were guided to read relevant content in the online survey explaining the objectives and significance of the study and requested to provide their digital signature. Only if they responded with ‘yes’ and informed consent was provided via a signature could they go to the questionnaire‐completion session. All data were handled in a private and non‐identifiable manner. Research ethics approval was obtained from the researcher's hospital where this study was conducted before data collection (The Approval No. was SCCHEC‐02‐2023‐035).

## 3 RESULTS

### 3.1 Demographic characteristics

In total, 390 questionnaires were completed, of which 349 were valid, with an effective return rate of 89.5%. Table 1 presents the participants' characteristics. Overall, 60.7% were 31–40 years old and 82.8% had received a bachelor's degree. Most participants were nurse practitioners (49.3%) and supervisor nurses (39.0%). Most (84%) worked in tertiary Grade A hospitals or in internal medicine (65%).

**TABLE 1:** Sample characteristics and their relationship with moral courage (*N* = 349).

Variables	*N* (%)	Mean ± SD of moral distress	*t*/*F*	*p* value
Sex			−1.007	0.315
Male	5(1.40%)	88 ± 8.66		
Female	344(98.60%)	81.32 ± 14.78		
Age(years)			8.594	<0.001*
≤30 years	82(23.50%)	77.68 ± 15.62		
31–40 years	212(60.70%)	81.15 ± 14.50		
≥41 years	55(15.80%)	81.42 ± 14.72		
Education level			16.896	<0.001*
Junior college	52(14.90%)	72.23 ± 10.39		
Bachelor degree	289(82.80%)	82.65 ± 14.71		
Master or above	8(2.30%)	96.75 ± 11.32		
Title			105.780	<0.001*
Primary title	188(53.80%)	73.26 ± 12.72		
Intermediate title	136(39.00%)	89.65 ± 1.67		
Senior title	25(7.20%)	98.00 ± 6.89		
Marital status			0.498	0.608
Unmarried	49(14.00%)	82.24 ± 15.43		
Married	282(80.80%)	81.09 ± 14.68		
Divorced/ widowed	18(5.20%)	84.33 ± 13.68		
The nature of your hospital			0.492	0.623
General hospital	237(67.90%)	81.68 ± 14.95		
Specialist hospital	112(32.10%)	80.86 ± 14.28		
The level of your hospital			3.385	0.018
Tertiary Grade A hospital	293(84.00%)	81.01 ± 14.57		
Tertiary Grade B hospital	31(8.90%)	78.65 ± 14.65		
Secondary Grade A hospital	15(4.30%)	87.60 ± 16.11		
Secondary Grade B hospitals and below	10(2.90%)	81.42 ± 14.72		
The category of your hospital			1.931	0.089
National hospital	21(6.00%)	80.67 ± 15.32		
Provincial hospital	110(31.50%)	78.92 ± 13.65		
Municipal hospital	157(45.00%)	83.59 ± 14.86		
County‐level hospital	42(12.00%)	78.88 ± 14.39		
District hospital	11(3.20%)	87.09 ± 18.47		
Other	8(2.30%)	80.88 ± 16.88		
Position			2.094	0.101
Common nurse	220(63.00%)	80.37 ± 15.57		
Leader nurse	85(24.40%)	81.57 ± 12.69		
The head nurse of a ward	35(10.00%)	86.63 ± 13.05		
The head nurse of a department	9(2.60%)	85.56 ± 16.09		
Department			3.368	0.019
Internal medicine	227(65.00%)	83.22 ± 14.35		
Surgery department	50(14.30%)	77.62 ± 13.86		
Department of radiotherapy	32(9.20%)	77.84 ± 13.41		
Emergency department and Intensive Care Unit	35(11.50%)	78.80 ± 17.33		
Employment form			0.254	0.776
Hospital employment	268(76.80%)	81.12 ± 15.16		
Government Employment	71(20.30%)	82.32 ± 12.94		
Other	10(2.90%)	83.10 ± 15.57		
Working experience in nursing			2.371	0.052
≤5 years	27(7.70%)	79.52 ± 13.52		
6–10 years	97(27.80%)	80.62 ± 16.10		
11–15 years	122(35.00%)	79.48 ± 15.24		
15–20 years	51(14.60%)	83.94 ± 13.97		
≥20 years	52(14.90%)	85.98 ± 13.19		
The time of having obtained the certificate of Oncology specialist nurse			42.275	<0.001*
<5 years	113(32.40%)	73.75 ± 13.11		
5–10 years	169(48.40%)	82.20 ± 14.30		
>10 years	67(19.20%)	92.39 ± 10.25		
The rank of certificate of oncology specialist nurses			0.120	0.904
National	25(7.20%)	81.08 ± 15.02		
Provincial	324(92.80%)	81.45 ± 14.72		
Religious belief			1.255	0.286
None	323(92.60%)	81.09 ± 14.22		
Buddhism	21(6.00%)	86.33 ± 21.02		
Other	5(1.40%)	82.00 ± 14.73		
Whether have received training in medical ethics			−5.671	<0.001*
No	117(33.50%)	75.39 ± 14.85		
Yes	232(66.50%)	84.46 ± 13.71		

* *p* < 0.001.

Nearly half of the participants (48.4%) reported that they had obtained oncology specialist nursing certification over the preceding 5–10 years and more than half of the nurses had received training in medical ethics. Specific demographic data are presented in Table 1.

### 3.2 Results of the bivariate analyses between demographic variables and moral courage

Bivariate analysis results suggested that age, educational level, title, level of hospital, department, time of obtaining certification and whether they received training in medical ethics were significantly associated with moral courage (*p* < 0.05). According to the results of the LSD test, participants with a mean age of over 41 years exhibited considerably stronger moral courage than those aged of less than 30 years (*p* < 0.001). Compared with junior college students, nurses with a master's degree or higher scored substantially higher (*p* < 0.001). Nurses who have senior title are substantially higher than primary title (*p* < 0.001). The scores of nurses employed in secondary Grade B and below hospitals were significantly lower than those of nurses working in tertiary Grade A hospitals (*p* < 0.05). Internal medicine nurses scored significantly higher (*p* < 0.05) than nurses working in surgery departments. Those with more than 10 years of experience working as oncology specialist nurses significantly outscored those with less than 5 years of experience (*p* < 0.001).

### 3.3 The relationship between moral courage, distress and sensitivity

The subscale scores for the three scales are presented in Table 2. First, we performed a correlation analysis to explore the relationships among moral courage, moral distress, and moral sensitivity. The results of the Pearson's correlation analyses are shown in Table 3. According to the results, moral courage had a weak and negative relationship with moral distress (*r* = −0.223, *p* < 0.01) and moral courage had a significant and positive relationship with moral sensitivity (*r* = 0.547, *p* < 0.01).

**TABLE 2:** The subscale scores of three scale.

Variable	Number of items	Score ranges	Mean ± SD
Moral distress scale	22	0–352	64.48 ± 48.99
Individual responsibility	8	0–128	20.92 ± 17.34
Not in the patients' best interest	5	0–80	15.00 ± 12.89
Value conflict	6	0–96	21.08 ± 16.63
Damage patients' interest	3	0–48	7.48 ± 7.25
Moral sensitivity scale	9	15–54	45.36 ± 6.43
Moral responsibility and strength	5	8–30	26.37 ± 3.57
Sense of moral burden	4	4–24	18.99 ± 3.70
Moral courage scale	21	21–105	81.42 ± 14.72
Compassion and true presence	5	5–25	19.71 ± 3.65
Moral responsibility	4	4–20	15.50 ± 3.11
Moral integrity	7	7–35	27.72 ± 4.98
Commitment to good care	5	5–25	18.48 ± 3.95

**TABLE 3:** Correlations among three scales (*N* = 349).

Variables	Moral integrity	Commitment to good care	Compassion and true presence	Moral responsibility
Individual responsibility	−0.229*	−0.210*	−0.231*	−0.201*
Not in the patients' best interest	−0.156*	−0.153*	−0.191*	−0.138*
Value conflict	−0.171*	−0.167*	−0.222*	−0.161*
Damage patients' interest	−0.180*	−0.164*	−0.211*	−0.168*
Moral responsibility	0.586*	0.547*	0.629*	0.584*
Sense of moral burden	0.311*	0.358*	0.305*	0.323*

* *p* < 0.001.

### 3.4 Results of the multiple linear regression

A multivariate linear regression analysis was performed with all statistically significant factors (age, educational level, title, level of hospital, department, time of obtaining certification, and whether they had received training in medical ethics) determined using the t‐test and ANOVA, with moral distress and moral sensitivity as independent variables, and moral courage as the dependent variable. The multiple linear regression results are presented in Table 4. The ANOVA results of the regression model showed that the linear regression equations fit the data well (*F* = 59.487, *p* < 0.001). The results indicated that having a master's degree or above, an intermediate or senior title, medical ethics training, moral distress and moral sensitivity contributed to explaining 54.1% of the variance in moral courage. In contrast to those with lesser qualifications, those with a master's degree or above had a significantly higher score for moral courage (*β* = 0.086, *p <* 0.05). Compared with having primary titles, having intermediate (*β* = 0.373, *p <* 0.001) and senior titles (*β* = 0.328, *p* < 0.001) showed a significantly positive influence on moral courage. Having received medical ethics training had a significantly positive effect on moral courage (*β* = 0.132, *p* < 0.001). Experiencing moral distress had a significantly negative influence on the likelihood of having moral courage (*β* = −0.138, *p* < 0.001) whereas moral sensitivity had a significant positive influence on moral courage (*β* = 0.356, *p* < 0.001).

**TABLE 4:** Multiple linear regression with predictor variables.

Variables	*B*	SE	*β*	*t*	*p*	95% *CI*
Constant	33.898	4.412		7.684	<0.001*	
Master degree or above	8.488	3.913	0.086	2.169	0.031	(0.790,16.185)
Intermediate title	11.229	1.266	0.373	8.866	<0.001*	(8.738,13.720)
Senior title	18.675	2.245	0.328	8.319	<0.001*	(14.259,23.090)
Whether having received medical ethic training	4.102	1.172	0.132	3.501	0.001	(1.797,6.407)
Moral sensitivity	0.816	0.090	0.356	9.034	<0.001*	(0.639,0.994)
Moral distress	−0.041	0.011	−0.138	−3.698	<0.001*	(−0.064, −0.019)

*Note*: *F* = 59.487, *p* < 0.001, *R* ^2^ = 0.541(adjusted).

Abbreviations: *B*, unstandardized regression coefficient; *CI*, confidence interval; SE, standard error; VIF, variance inflation factor; *β*, standardized regression coefficient.

* *p* < 0.001.

## 4 DISCUSSION

This study presented an overview of moral courage among Chinese oncology specialist nurses. To the best of our knowledge, this study is the first to investigate the relationships between moral courage, moral distress and moral sensitivity among oncology specialist nurses. This study provides new insights into moral courage and the benefits of relieving moral distress and enhancing ethical safety.

Considerable empirical research has been conducted to better understand nurses' moral courage and how to support them under complex ethical challenges. Previous cross‐sectional studies have explored the relationship among nurses (Gibson et al., 2020; Hauhio et al., 2021; Hu et al., 2022; Peng et al., 2023; Wenwen et al., 2018). One study demonstrated that the mean score for moral courage among nurses working in ICUs in COVID‐19 pandemic hospitals was 82.08 (Goktas et al., 2023). The average score for moral courage among all types of nurses was 72.09 (Ali Awad & Al‐Anwer Ashour, 2022). The results of these studies indicated that the average score for moral courage among intensive care nurses was slightly higher than that among oncology specialist nurses, and significantly higher than that for all types of nurses. This may be because patients in ICUs have a higher probability and quicker rate of death. As a result, intensive care specialist nurses have deeper knowledge and experience in dealing with complex scenarios than other types of nurses. These reasons may explain the relatively lower level of moral courage among oncology specialist nurses compared to intensive care nurses. Given the importance of moral courage, the results indicate the urgency of initiating a moral courage training programme for nurses.

Importantly, we found that the level of moral courage among oncology specialist nurses was associated with their educational level, title, and whether they had received training in medical ethics. Educational level is widely recognized as a significant factor in relation to moral courage (Goktas et al., 2023; Konings et al., 2022; Pirdelkhosh et al., 2022). With more advanced levels of education, nurses gain a stronger ability to cope with complex situations and their level of moral courage is enhanced. Age was shown to have no relationship with moral courage, which contradicts the findings of previous studies (Hauhio et al., 2021; Konings et al., 2022; Wenwen et al., 2018). Title was identified as a significant influencing factor in relation to moral courage in our study, although this finding differs from that reported in another recent study (Huang et al., 2023). We consider that nurses with high‐level professional titles generally have more comprehensive personal abilities, richer nursing knowledge and skills, and can better deal with clinically complex situations or moral dilemmas; therefore, they would have higher moral courage. Receiving training in medical ethics was found to be a significant factor in relation to moral courage, similar to the findings of a previous study (Alshammari & Alboliteeh, 2023). Nurses who received training in medical ethics had a higher level of moral courage than other nurses, which is most likely explained by other nurses obtaining a more limited understanding of moral evaluations in general training, whereas training focused on moral knowledge would have quickly enabled such nurses to have become better informed. All the four demographic characteristics were closely related. With higher levels of educational level, title, and medical ethics‐related training, relevant knowledge could be expected to increase along with nurses' ability to identify moral conflict situations and for their moral courage to develop positively, with a subsequent decrease in the influence of moral distress on nurses' work environments.

There is consensus that moral courage has a positive relationship with moral sensitivity (Escolar‐Chua, 2018) and a negative relationship with moral distress (Escolar‐Chua, 2018; Peng et al., 2023). Nurses with high levels of moral courage view moral distress positively and are more willing to discuss ethical problems encountered with relevant professionals, promote the development of professional relations of mutual respect and cooperation, reduce moral conflict in their environments, and create a good ethical atmosphere to reduce moral distress. However, moral sensitivity is positively related to moral courage. Due to their moral sensitivity, nurses tend to hold to their ethical standards and exhibit courage when helping patients with cancer confront ethical problems or patients with incomplete self‐awareness when performing their nursing duties.

These findings show that moral courage is required in various care contexts. Seeking unprofessionally to modify the behaviour of other professionals, misapplying ethical evaluations derived from other professional domains to that of nursing, and adapting simplistic conventionalism in ethical decision‐making have all been linked to a lack of moral courage (Hauhio et al., 2021). Education substantially influences the moral courage of nurses, with individuals being able to acquire relevant ethical principles through moral education (Begum et al., 2022). Effective moral education should catalyse societal change and promote the development of a moral consciousness (Begum et al., 2022). Various educational intervention studies and their impact on and effectiveness in building nurses' moral courage at all organizational and educational levels should be taken into consideration (Hauhio et al., 2021). Simulations and vicarious learning (Hauhio et al., 2021; Roberts, 2010), digital stories (LeBlanc, 2017), ethics workshops (Beumer, 2008) and narrative writings (Tsuruwaka & Asahara, 2018) are important methods for enhancing moral education. The evaluation of teaching materials, instructional strategies and the evaluation method itself should be primary topics in educational research (Hauhio et al., 2021).

To date, this is the first study to investigate the moral courage of oncology specialist nurses and its influencing factors in China. Nursing practice involves intrinsic ethical challenges, which affect teams, companies, families, patients and nurses personally (Rushton, 2023). This study provides insights for improving moral courage and sensitivity. First, the results have implications for clinical practice. This study indicates that there is still room for enhancing moral courage, which may serve as a reference for policymakers and stakeholders to promote an ethical environment and ensure that nursing is implemented ethically. For example, internal and organizational limitations, an increase in workload, and a lack of support that affect nurses' physical and mental well‐being and intention to leave are all elements that contribute to moral distress (Edwin et al., 2023). Hospital administrations need to focus on improving rules and regulations to boost moral courage and mitigate moral distress, which would contribute to enhanced clinical competence and skills application (Kalani et al., 2023).

In addition, because of the vast territory and numerous nationalities in China, studies need to focus on different types of interventions to explore effective methods for enhancing moral courage in different research contexts, especially in terms of the various regions and hospitals. Nursing managers should propose customized management plans based on the types of moral courage identified as necessary within the nursing community and the coping styles required in different contexts (Hong et al., 2023). Second, the results demonstrated that receiving relevant medical ethics training was an important controllable variable. Moral education is a critical method to help face challenges in that it can help improve moral courage and moral sensitivity and further hinder the occurrence of moral distress. In keeping with care ethics, this type of action is necessary to ensure the well‐being of patients by acknowledging their specific interests and preferences (Milliken & Grace, 2017). Medical schools are recommended to set up specialist courses to strengthen basic learning about nursing ethics and hospitals should attach more importance to embedding ethics education into continuous courses. Effective teaching strategies in moral education include involving students in the teaching process, providing opportunities for practice and experience, practicing simulated scenarios and repeating lessons (Jahangasht Ghoozlu et al., 2023).

### 4.1 Limitations

This study had some limitations. First, China has a vast territory with 34 provinces and 56 nationalities, and as a result, nurses have different professional abilities. Owing to a lack of corresponding resources, we surveyed only 15 cities in Sichuan Province, which may limit the generalizability of the findings. Second, nurses working in tertiary Grade A hospitals were mainly recruited for the study. Nurses working in primary healthcare institutions need to be focused on in future research. Third, as this was a cross‐sectional study, causal relationships could not be determined and further longitudinal studies are recommended to verify our results.

## 5 CONCLUSION

The study results indicate that having a master's degree or above, an intermediate or senior title, medical ethics training, moral distress and moral sensitivity had a significant association with moral courage and that there is room for improvement in moral courage among Chinese oncology specialist nurses through adopting appropriate training strategies and support. Policymakers and stakeholders should develop clinical intervention strategies in different research contexts to promote ethical environments and ensure that nursing is implemented ethically. In addition, we proposed effective methods to perfect moral education in training schools and in continuous education.

## AUTHOR CONTRIBUTIONS

All authors contributed to the study's conception and design. Naifang Xing, Hui Huang and Yanling Cai conducted data collection. Data analysis was performed by Yao Cen and Xiaohui Tan. The first draft of the manuscript was written by Hongling Zheng and subsequently revised by Lei Luo and Xiaohui Tan. All authors commented on previous versions of the manuscript. All authors read and approved the final manuscript.

## FUNDING INFORMATION

This study was supported by the 2023 Sichuan cadre health research project (2023–806).

## CONFLICT OF INTEREST STATEMENT

No potential conflicts of interest were disclosed by the author(s) concerning the research, writing, or publication of this paper.

## Data Availability

The data that support the findings of this study are available from the corresponding author upon reasonable request.
